# The “sandwich” procedure for paracentral rheumatoid corneal perforation: a case report with 6-year follow-up and literature review

**DOI:** 10.3389/fmed.2025.1649417

**Published:** 2025-10-10

**Authors:** Yi Zhang, Hanjing Liu, Qing Li, Yan Zhu, Yu-guang Zhu

**Affiliations:** ^1^Shandong Second Medical University, Weifang, China; ^2^Eye Center, The Affiliated Hospital of Shandong Second Medical University, Weifang, China

**Keywords:** “sandwich” procedure, case report, corneal perforation, rheumatoid arthritis, intrastromal allograft keratoplasty

## Abstract

Rheumatoid arthritis (RA) is a chronic systemic autoimmune disease often associated with ocular manifestations. In rare cases of RA, paracentral rheumatoid corneal perforation may occur. We report the case of a 53-year-old monocular woman with a 20-year history of RA who presented to the clinic with a paracentral corneal perforation. Slit-lamp examination revealed a 2-mm diameter paracentral perforation with an iris plug. The patient was clinically diagnosed with sterile rheumatoid corneal perforation. We describe an innovative “sandwich” procedure developed for addressing the corneal perforation. Initially, a partial thickness limbal groove was created outside the perforation, followed by the formation of a semicircular intrastromal pocket extending approximately 2 mm inside the perforation edge. A lamellar graft was then fashioned and inserted into the intrastromal pocket. Subsequently, the limbal groove was closed, and a conjunctival flap was used to cover the perforated area. Upon follow-up, the “sandwich” procedure provided sufficient tectonic support for the patient’s only eye, resulting in a stable ocular surface. Over a 6-year follow-up period, the postoperative best-corrected visual acuity (BCVA) was maintained at 20/50. To the best of our knowledge, this is the first report of the “sandwich” procedure for paracentral rheumatoid corneal perforation.

## 1 Introduction

Rheumatoid arthritis (RA) is a prevalent systemic autoimmune disorder, often associated with various ocular manifestations. These include severe dry eye syndrome (aqueous tear deficiency), Sjögren’s syndrome, peripheral ulcerative keratitis, scleritis, and corneal melting, all of which have been extensively documented (1).

Although paracentral rheumatoid corneal perforation is a rare complication in RA patients, it poses significant risks, including severe anatomical distortion of the eye and consequent visual impairment (2, 3). In the most severe cases, corneal perforation can lead to blindness. Prompt surgical intervention is crucial for managing corneal perforations (4). However, the scarcity of fresh corneal tissue presents a significant challenge in treatment (5).

In this report, we present a novel “sandwich” technique for addressing a paracentral corneal perforation (2 mm in diameter) in a monocular patient with rheumatoid arthritis. The procedure involved creating a semicircular intrastromal pocket and inserting a sandwiched patch into it. The technique was further reinforced with a conjunctival flap.

## 2 Case presentation

A 53-year-old monocular female with a 20-year RA history presented to the clinic with a paracentral corneal perforation. Her ophthalmic history indicated complaints of pain and a foreign body sensation in her left eye, with corneal ulceration developing 1 month ago. Despite ongoing treatment with topical 0.5% ofloxacin eyedrops administered six times daily, she was referred to the clinic due to a sudden vision decrease in her only eye. The best-corrected visual acuity (BCVA) was measured at 20/200 in the left eye, with the right eye having been enucleated. Slit-lamp examination identified a 2-mm diameter inferonasal paracentral perforation with an iris plug and a shallow anterior chamber was observed. The clinical diagnosis was sterile paracentral rheumatoid corneal perforation.

Subsequently, an intrastromal allograft keratoplasty combined with a conjunctival flap, referred to as the “sandwich” procedure, was performed on the affected eye the following day.

### 2.1 Surgical procedure

**Figure 1:**
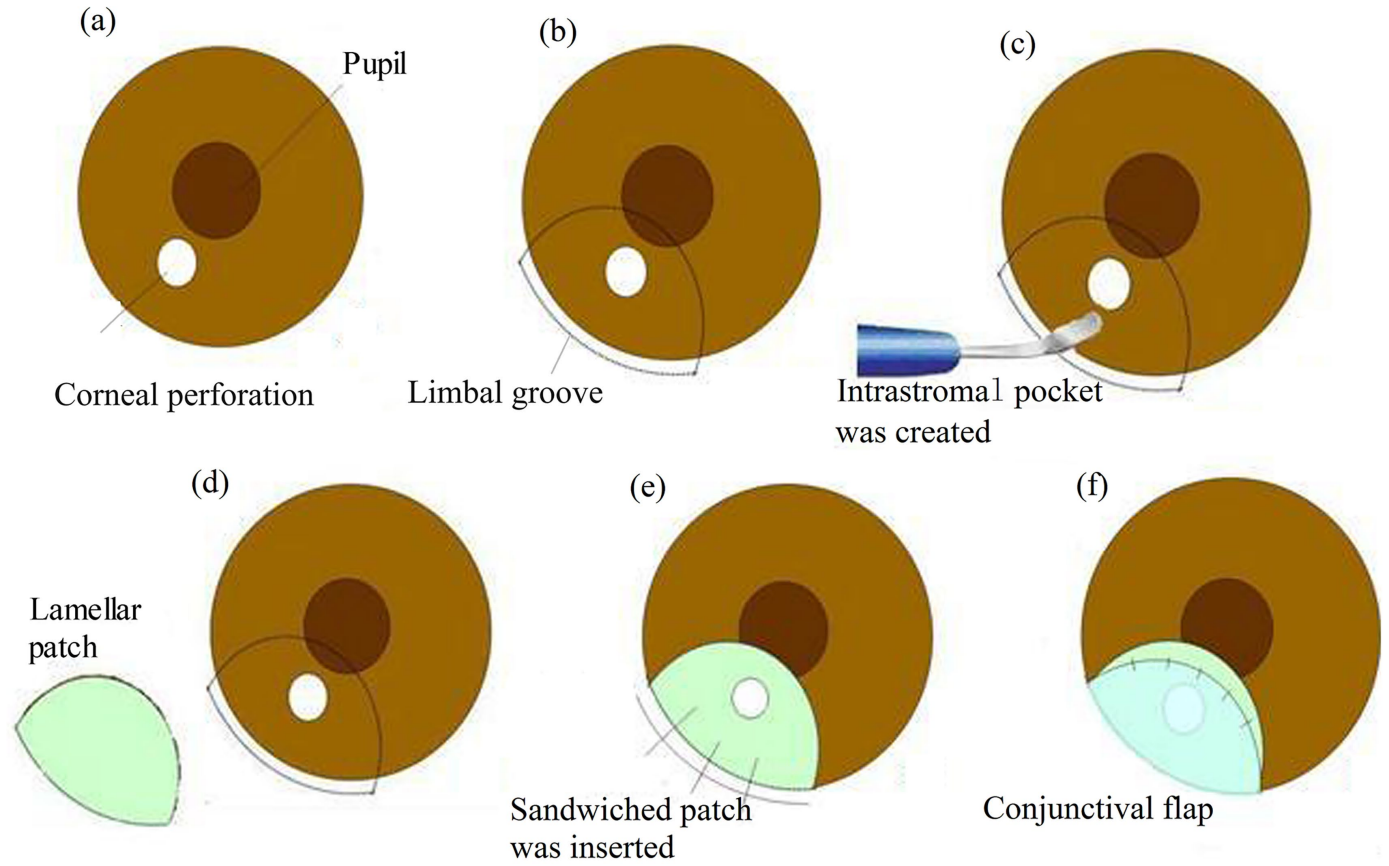
The “sandwich” procedure. **(a)** A paracentral corneal perforation. **(b)** The demarcation of the perforated corneal area (ensure a margin of at least 2 mm from the inner edge of the perforation) in a semicircular configuration. **(b)** A partial thickness limbal groove (radius approx. 6.0 mm) was made outside the perforation. **(c)** The corneal intrastromal pocket was created using a crescent knife. **(d)** The lamellar graft (radius approx. 5.5 mm) was cut in a semicircular shape. **(e)** The sandwiched patch (thickness approx. 220–250 μm) was inserted into the designed intrastromal pocket and the groove was secured with 10-0 mono-filament nylon sutures. **(f)** Conjunctival flap covered the perforated area of the cornea.

The “sandwich” procedure is detailedly shown in Figure 1.

Following the careful demarcation of the perforated corneal area to ensure a margin of at least 2 mm from the inner edge of the perforation in a semicircular configuration. A limbal groove with a thickness of 400 mm was created outside the perforation using a diamond knife.

Subsequently, a side incision was made with a stab knife. Sodium hyaluronate (15 mg/mL) was then injected into the anterior chamber to release the pluged iris.

A semicircular intrastromal pocket was fashioned, extending approximately 2 mm inside the perforation edge, using a crescent knife. A lamellar patch dissection of the donor tissue was performed to remove the epithelium, endothelium, and anterior stroma. The lamellar graft was shaped into a semicircular form, slightly smaller than the intrastromal pocket, and was gently inserted into the pocket. The groove was then securely closed with three interrupted 10-0 monofilament nylon sutures.

In this case, a conjunctival flap was necessary to cover the perforation area. This flap, away from the visual axis, was affixed to the healthy cornea with five interrupted 10-0 nylon sutures. The suture knots were buried.

Finally, the sodium hyaluronate was thoroughly washed out, and the side incision was checked for water tightness.

Postoperatively, the patient was prescribed topical 0.5% levofloxacin and 1% ciclosporin eyedrops four times daily for 1 month, followed by 1% ciclosporin eye drops four times daily for a further 2 months. The 1% ciclosporin was then gradually tapered to once daily over several months. Currently, maintenance therapy with topical 0.4% hyaluronic acid is being administered.

**Figure 2:**
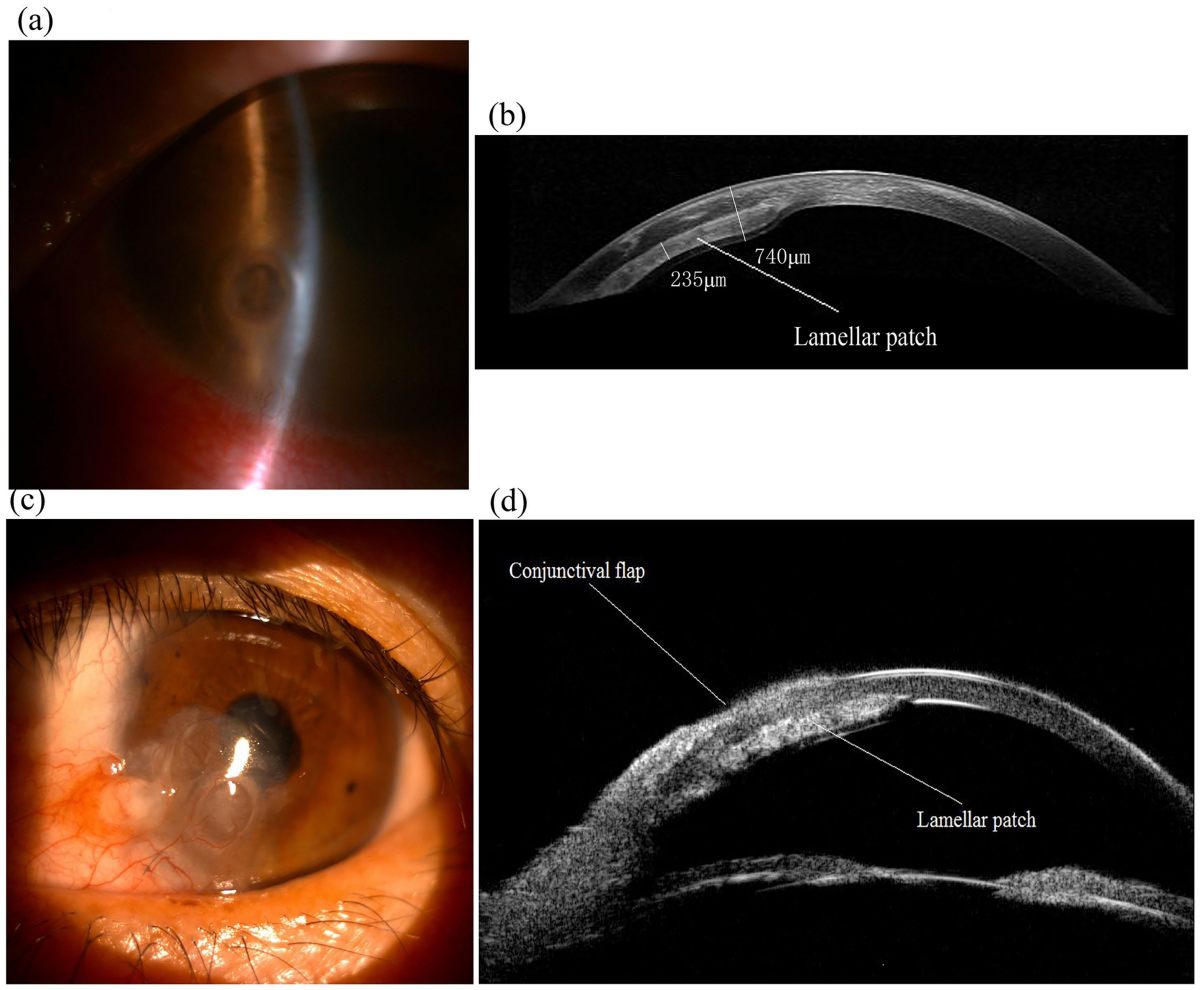
Images of the only eye of an 53-year-old female patient with a paracentral corneal perforation and performed the “sandwich” procedure. **(a)** Preoperative slit-lamp photograph showed a 2-mm diameter inferonasal paracentral perforation and iris plug in the only eye. **(b)** Anterior segment OCT imaging of the eye 6 years after the “sandwich” procedure, displaying lamellar patch (sandwiched patch) remains in position (thickness approx. 220–250 μm). **(c)** Slit-lamp photograph of the eye 6 years after surgery, showing the eye maintains a stable ocular surface. **(d)** UBM imaging of the eye 6 years after surgery showed the “sandwich” configuration.

Follow-up included physical examinations, anterior segment optical coherence tomography (OCT), and ultrasound biomicroscopy (UBM) scans (Figure 2). No intraoperative or postoperative complications were observed, and the ocular surface remained stable. The postoperative corrected distance visual acuity (CDVA) was 20/50 in the only eye at 6 months and remained stable over a 6-year follow-up period.

## 3 Discussion

Corneal perforations can result in various degrees of ocular tissue damage and visual impairment (2). The predominant cause of corneal perforation is microbial infection (3), which is responsible for most central corneal perforations. In contrast, peripheral corneal perforations are primarily secondary to degeneration, autoimmune diseases, and microbial infections. The topical application of certain medications, including antibiotics, corticosteroids, and nonsteroidal anti-inflammatory drugs (NSAIDs), may initiate or exacerbate corneal melting, potentially leading to corneal perforation (6).

This report described the case of paracentral corneal perforation in a RA patient. But unknown causes were responsible for the perforation without history of ocular infection or trauma in the only eye. Aqueous tear deficiency, severe dry eye, keratolysis, and superficial corneal ulceration associated with RA may have contributed to the corneal perforation (7, 8).

Management of corneal perforation necessitates a tailored approach based on the perforation’s status and the patient’s medical history. Bandage soft contact lenses, tissue glue (9), medication administration (10), amniotic membrane transplantation (AMT) (11), descemet stripping automated endothelial keratoplasty (DSAEK) (12), and conjunctival flaps (13) are not suitable for the large corneal perforations (≥2 mm diameter), which require therapeutic keratoplasty (1).

Penetrating keratoplasty (PKP) is a predominant surgical intervention for addressing corneal perforation, aimed at preserving the structural integrity of the globe and restoring visual function (14). However, the scarcity of donor corneas poses a significant challenge to the widespread implementation of PKP, particularly in developing countries such as China (15).

The shortage of fresh donor tissue has led to the utilization of cryopreserved corneas for treating corneal perforation. In emergency situations, lamellar keratoplasty (LKP) may serve as an effective procedure for peripheral corneal perforation (16). In cases of central corneal perforation, LKP is considered a temporary measure, with the expectation that subsequent transplantation with fresh donor tissue will yield superior visual outcomes.

Intralamellar autopatch was used for paracentral corneal perforation (17). The procedure was reported to avoid the central sutures into the visual axis. However, the larger surgical wound, graft-host junctional opacification and peripheral suture-induced astigmatism hinder substantial visual improvement.

As a lamellar graft, standard DSAEK was performed for corneal perforation (12). The technique avoided suture-related complications and encroachment of the graft-host junction. However, DSAEK remained limited in most cases of corneal perforation for endothelial immune rejection, corneal endothelium damage, and scarcity of fresh donor corneas.

Although intrastromal LKP has demonstrated potential efficacy in treating pellucid marginal degeneration (PMD) (18), its use in addressing corneal perforation has not been previously documented.

In this report, we introduce the “sandwich” procedure for managing paracentral corneal perforation (2 mm in diameter) using cryopreserved donor tissue. Intrastromal LKP provided sufficient tectonic support, suggesting its potential utility in treating corneal perforation, particularly in patients with severe ocular surface diseases such as RA, where therapeutic PKP often yields suboptimal outcomes. Additionally, a conjunctival flap was employed to facilitate corneal healing in this case.

The intrastromal lenticule obtained from small-incision lenticule extraction (SMILE) procedure was used as a patch graft in LKP to address corneal thinning and perforation (19, 20). This approach partially mitigates the scarcity of fresh donor corneas. Single-layer lenticule is insufficiently thick for use in the “sandwich” technique. However, double-layer lenticules may prevent bulging and protrusion of the patch graft within the lamellar plane with more complex procedure (19).

The procedure effectively preserved the transparency of the visual axis and improved visual acuity. This method provides a simple way to close sterile paracentral corneal perforations without requiring intraocular intervention. For peripheral corneal perforation, intrastromal tamponade using a lamellar allograft may be a viable option.

Imaging assessments, including anterior segment optical coherence tomography (OCT) and ultrasound biomicroscopy, showed that the globe was intact and that the donor graft was providing sufficient tectonic support after a 6-year postoperative follow-up.

The “sandwich” technique presents several limitations. Firstly, the overlap of the surgical area with the pupil and the thickening of the recipient cornea resulted in the irregular astigmatism, potentially limited visual improvement. Secondly, the “sandwich” technique may not be suitable for addressing central perforations or large paracentral perforations exceeding 3 mm in diameter. Thirdly, the conjunctival flap increases the risk of corneal vascularization (15), it may facilitate corneal healing and offer more advantages than disadvantages for this patient with a long history of rheumatoid arthritis.

## 4 Conclusion

We introduce an innovative “sandwich” technique for the management of paracentral corneal perforation utilizing cryopreserved donor tissue. This “sandwich” procedure successfully provided adequate tectonic support for the patient’s sole functional eye, leading to the stabilization of the ocular surface. This case indicates the potential applicability of this approach in the treatment of corneal perforation, especially in patients with severe ocular surface diseases, such as RA, where therapeutic PKP frequently results in suboptimal outcomes.

## Data Availability

The original contributions presented in the study are included in the article/supplementary material, further inquiries can be directed to the corresponding authors.

## References

[1] Artifoni M Rothschild PR Brézin A Guillevin L Puéchal X. Ocular inflammatory diseases associated with rheumatoid arthritis. Nat Rev Rheumatol. (2014) 10:108–16. doi: 10.1038/nrrheum.2013.185, PMID: 24323074

[2] Timlin HM Hall HN Foot B Koay P. Corneal perforation from peripheral ulcerative keratopathy in patients with rheumatoid arthritis: epidemiological findings of the British Ophthalmological Surveillance Unit. Br J Ophthalmol. (2018) 102:1298–302. doi: 10.1136/bjophthalmol-2017-310671, PMID: 29246891

[3] Abd Elaziz MS Zaky AG El SaebaySarhan AR. Stromal lenticule transplantation for management of corneal perforations; one year results. Graefes Arch Clin Exp Ophthalmol. (2017) 255:1179–84. doi: 10.1007/s00417-017-3645-6 28409225

[4] Jhanji V Young AL Mehta JS Sharma N Agarwal T Vajpayee RB. Management of corneal perforation. Surv Ophthalmol. (2011) 56:522–38. doi: 10.1016/j.survophthal.2011.06.003, PMID: 22117886

[5] Gain P Jullienne R He Z Aldossary M Acquart S Cognasse F. Global survey of corneal transplantation and eye banking. JAMA Ophthalmol. (2016) 134:167–73. doi: 10.1001/jamaophthalmol.2015.4776, PMID: 26633035

[6] Wolf EJ Kleiman LZ Schrier A. Nepafenac-associated corneal melt. J Cataract Refract Surg. (2007) 33:1974–5. doi: 10.1016/j.jcrs.2007.06.043, PMID: 17964407

[7] Wajnsztajn D Nche E Solomon A. Corneal complications of rheumatoid arthritis. Curr Opin Allergy Clin Immunol. (2022) 22:304–13. doi: 10.1097/ACI.0000000000000844, PMID: 35980013

[8] Shah R Amador C Tormanen K Ghiam S Saghizadeh M Arumugaswami V. Systemic diseases and the cornea. Exp Eye Res. (2021) 204:108455. doi: 10.1016/j.exer.2021.108455, PMID: 33485845 PMC7946758

[9] Khalifa YM Bailony MR Bloomer MM Killingsworth D Jeng BH. Management of nontraumatic corneal perforation with tectonic drape patch and cyanoacrylate glue. Cornea. (2010) 29:1173–5. doi: 10.1097/ICO.0b013e3181d5d996, PMID: 20622669

[10] Nahum Y Bahar I Busin M. Tectonic descemet stripping automated endothelial keratoplasty for the management of sterile corneal perforations in decompensated corneas. Cornea. (2016) 35:1516–9. doi: 10.1097/ICO.0000000000001037, PMID: 27749446

[11] Namba H Narumi M Nishi K Goto S Hayashi S Yamashita H. “Pleats fold” technique of amniotic membrane transplantation for management of corneal perforations. Cornea. (2014) 33:653–7. doi: 10.1097/ICO.0000000000000128, PMID: 24763120

[12] Smyth A McCabe GA Murtagh P McElnea EM. Tectonic descemet’s stripping automated endothelial keratoplasty for corneal perforation. BMJ Case Rep. (2022) 15:e247345. doi: 10.1136/bcr-2021-247345 PMC9036169 35459648

[13] Sandinha T Zaher SS Roberts F Devlin HC Dhillon B Ramaesh K. Superior forniceal conjunctival advancement pedicles (SFCAP) in the management of acute and impending corneal perforations. Eye. (2006) 20:84–9. doi: 10.1038/sj.eye.6701814, PMID: 15803178

[14] Yang Y Zeng H Gong L Lin T. Risk factors for graft failure after penetrating keratoplasty in eastern China from 2018 to 2021. Ann Transplant. (2024) 29:e945388. doi: 10.12659/AOT.945388, PMID: 39434378 PMC11512512

[15] Shekhawat NS Kaur B Edalati A Abousy M Eghrari AO. Tenon patch graft with vascularized conjunctival flap for management of corneal perforation. Cornea. (2022) 41:1465–70. doi: 10.1097/ICO.0000000000003068, PMID: 36219216 PMC9558081

[16] Huang T Wang Y Ji J Gao N Chen J. Evaluation of different types of lamellar keratoplasty for treatment of peripheral corneal perforation. Graefes Arch Clin Exp Ophthalmol. (2008) 246:1123–31. doi: 10.1007/s00417-008-0812-9, PMID: 18446359

[17] Titiyal JS Ray M Sharma N Sinha R Vajpayee RB. Intralamellar autopatch with lamellar keratoplasty for paracentral corneal perforations. Cornea. (2002) 21:615–8. doi: 10.1097/00003226-200208000-00019 12131044

[18] Jabbarvand M Hashemian H Khodaparast M Hassanpour N Mohebbi M. Intrastromal lamellar keratoplasty in patients with pellucid marginal degeneration. J Cataract Refract Surg. (2015) 41:2–8. doi: 10.1016/j.jcrs.2014.11.030, PMID: 25532629

[19] Pant OP Hao JL Zhou DD Pant M Lu CW. Tectonic keratoplasty using small incision lenticule extraction-extracted intrastromal lenticule for corneal lesions. J Int Med Res. (2020) 48:300060519897668. doi: 10.1177/0300060519897668, PMID: 31975635 PMC7113716

[20] Tawfeek MMM Ahmed HMAH Bor’i A Rady AMNA. SMILE lenticule versus amniotic membrane graft (AMG) augmented with platelet-rich plasma (PRP) for the treatment of perforated corneal ulcer. Int Ophthalmol. (2023) 43:2341–8. doi: 10.1007/s10792-023-02631-3, PMID: 36692698

